# Spatiotemporal patterns and incidence trends of other infectious diarrhea in Qinghai Province, 2009–2023: a surveillance-based analysis

**DOI:** 10.3389/fpubh.2026.1776783

**Published:** 2026-04-21

**Authors:** Jinhua Zhao, Yuqi Jiang, Jiang Long, Ping Deng, Shenglin Qin, Yang Zhang

**Affiliations:** 1 Department of Public Health, Medical College of Qinghai University, Xining, China; 2 Business Department, Qinghai Provincial Center for Disease Control and Prevention, Xining, China; 3 Chongqing Municipal Academy of Preventive Medicine, Liangjiang New Area, Chongqing, China

**Keywords:** epidemiological characteristics, incidence rate, joinpoint regression, other infectious diarrhea, spatial analysis

## Abstract

**Background:**

While other infectious diarrhea (OID) has a persistent increase in occurrence throughout China, its epidemiological traits in high-altitude regions are inadequately studied. Qinghai Province, situated on the Tibetan Plateau, offers an essential context for analyzing OID patterns under unique climatic conditions.

**Methods:**

We examined reported incidence data for OID in Qinghai Province from 2009 to 2023. Temporal trends were evaluated by Joinpoint regression and Seasonal-Trend decomposition using Loess (STL) decomposition, while methods including spatial autocorrelation, cluster analysis, hotspot analysis, the Gravity center shift model, and standard deviation ellipse were utilized.

**Results:**

Between 2009 and 2023, there were 58,717 reported instances of OID, including one death in 2020. The overall incidence rate increased significantly, with an average annual percent change (AAPC) of 7.11% (95% CI: 2.03%−13.43%, *p* = 0.008). The escalation was more pronounced in females (AAPC = 9.11%, 95% CI: 1.03%−17.83%, *p* = 0.026) than in males (AAPC = 5.94%, 95% CI: −2.26% to 14.83%, *p* = 0.160). Substantial regional variations were apparent; although the highest incidence rates were located in Xining City and Haidong City, the fastest expansion transpired in Haibei City (AAPC = 15.71%, *p* < 0.001). A bimodal age distribution was discovered, with the highest incidence among children under 5 years (890.45 per 100,000) and the older adults, and the fastest escalation observed among young children. Spatial analysis indicated substantial positive spatial autocorrelation, with Moran's *I* values ranging from 0.404 to 0.643 (all *p* < 0.001), pinpointing Xining City and Haidong City as the principal high-high cluster areas encompassing eight counties. Standard deviational ellipse analysis revealed a primary clustering region centered around Xining and Haidong with a dominant northeast-southwest spatial axis. The incidence centroid remained consistently concentrated in the eastern area south of Xining city, exhibiting a three-phase migration pattern of stable fluctuation, significant southwestward shift, and gradual return to the core area over the study period.

**Conclusions:**

The incidence of OID in Qinghai Province has markedly risen, exhibiting distinct spatial clustering and demographic variations. These ecological findings suggest priority regions (Xining, Haidong) and populations (children under five, the older adults) for targeted public health interventions and warrant further investigation into environmental and healthcare-related determinants.

## 1 Introduction

Other infectious diarrhea (OID) is designated as a Class C notifiable infectious disease in China, encompassing diarrheal disorders induced by microorganisms distinct from those causing cholera, bacillary dysentery, typhoid, paratyphoid, and amebic dysentery (1, 2). From 2004 to 2017, the average annual incidence of OID in the Chinese Mainland was 60.64/100,000, especially in the Chinese Mainland since 2006 (APC = 4.12, 95% CI: 2.06–6.21) (3). In Fujian Province, a study reported 388,636 OID cases during 2005–2021 with an average annual incidence of 60.3 per 100,000, demonstrating continuous increases, seasonal shifts, and associations with meteorological factors, with children under 2 years accounting for 50.7% of cases, while similar increasing trends have been documented in Zhejiang Province, underscoring the escalating burden to regional public health systems (4, 5).

Diarrheal disorders continue to pose a significant global health challenge, especially impacting young children in Asia and Africa (6). They substantially impact global mortality, with an estimated 1.17 million deaths attributable to diarrheal diseases in 2021, and remain among the leading causes of death in children under 5 years. Prevalent risk factors for diarrheal diseases include contaminated water and inadequate sanitation; additionally, low birth weight has been identified as a risk factor predisposing children to more severe or frequent diarrheal infections, rather than a direct consequence of diarrheal disease itself (7). Moreover, case management and monitoring systems for diarrhea are frequently deficient in resource-constrained environments, highlighting an urgent necessity for enhancement (8–11). Consequently, performing comprehensive research on the epidemiological surveillance and control techniques of OID is of considerable public health significance. The sustained upward trajectory of OID incidence observed in recent years underscores the critical importance of a dedicated regional analysis. This research is crucial for formulating effective, context-specific strategies to diminish morbidity and death (12).

In high-altitude regions like Qinghai, where geographic seclusion and uneven allocation of healthcare resources can exacerbate the disease load, this necessity is particularly pronounced (13–15). Environmental mechanisms specific to high-altitude settings may influence disease transmission, including hypoxia-induced alterations in intestinal immunity, limited sanitation infrastructure due to permafrost conditions, and climatic constraints on pathogen survival (16, 17). However, these hypothesized mechanisms require further investigation. This research is crucial for formulating effective, context-specific strategies to diminish morbidity and death (18).

Despite the availability of national and regional OID surveillance studies focusing on eastern coastal provinces, comprehensive investigations in high-altitude regions remain limited. Existing studies in high-altitude areas have primarily focused on specific pathogens or short-term outbreaks, with no long-term spatiotemporal analysis of OID integrating temporal decomposition and advanced spatial methods. Furthermore, previous research has not systematically examined the spatial clustering patterns and centroid migration dynamics of OID in the Tibetan Plateau context. This study addresses these gaps by providing a long-term spatiotemporal analysis of OID in Qinghai Province, a representative high-altitude region on the northeastern Tibetan Plateau, combining Joinpoint regression, seasonal-trend decomposition, spatial autocorrelation analysis, standard deviational ellipse, and gravity center shift modeling to identify critical populations, high-risk areas, and spatial evolution patterns for region-specific surveillance and prevention strategies.

## 2 Materials and methods

### 2.1 Study areas

Qinghai Province is situated on the northeastern Tibetan Plateau in western China, covering approximately 720,000 square kilometers with an average elevation exceeding 3,000 meters (19, 20). The climate is continental and arid, characterized by long, cold winters, short cool summers, and low annual precipitation. Other infectious diarrhea consistently ranks among the top five notifiable infectious diseases in Qinghai, with children under 5 years showing particularly high incidence rates. The unique high-altitude environment, cold climate, and uneven healthcare resource distribution across the province underscore the need for region-specific OID research to inform targeted prevention strategies (21).

### 2.2 Data sources

Incidence data were sourced from the National Notifiable Infectious Disease Surveillance System under the China Information System for Disease Control and Prevention. Cases are mandatorily reported by all hospitals and community health centers via the web-based National Notifiable Infectious Disease Reporting System. Reporting is required for both clinically diagnosed cases (based on symptoms and epidemiological history) and laboratory-confirmed cases. The system undergoes routine quality control checks at provincial and national levels, including monthly data verification and annual completeness assessments.

Using “incidence date” and “current address” as query criteria, we extracted monthly and annual OID data for all 45 counties in Qinghai Province from January 2009 to December 2023. No specific ICD codes were used, as the surveillance system employs a standardized case classification system based on clinical and laboratory criteria. All reported OID cases meeting the national case definition during the study period were included; no exclusions were applied. The surveillance data were complete with no missing values for any county or year.

Annual age-stratified population denominators were obtained directly from the Qinghai Provincial Bureau of Statistics; no intercensal population estimates were used. All incidence rates reported in this study are crude rates, calculated as the number of reported cases divided by the corresponding population denominator, multiplied by 100,000. Age-standardized rates were not calculated, as the primary objective was to describe actual disease burden and trends in the population for public health planning purposes. The map data were supplied by the Qinghai Provincial Institute of Geological Survey. The map utilized in this study is scaled at 1:100,000,000 and accurately illustrates the governmental divisions of the region.

The research data constitute routine anonymized surveillance information in the field of public health. During both collection and storage phases, the data underwent de-identification processing in compliance with national public health data management protocols. All personally identifiable information was completely removed, with only aggregated data—specifically county-level geographical divisions and disease incidence counts—being retained. Consequently, no risk of personal privacy breach exists. In accordance with China's “Ethical Review Measures for Biomedical Research Involving Humans,” ethical review and informed consent are therefore waived.

### 2.3 Case definition

According to the national surveillance protocol issued by the National Health Commission of the People's Republic of China (WS 271-2007), OID is defined as diarrheal diseases caused by bacteria, viruses, parasites, or fungi, excluding pathogens responsible for cholera, bacillary dysentery, typhoid, paratyphoid, and amebic dysentery, encompassing both clinically diagnosed cases (≥3 loose or watery stools per 24 h) and laboratory-confirmed cases with identified pathogens, with cases mandatorily reported by all hospitals and community health centers via the web-based National Notifiable Infectious Disease Reporting System (13). Throughout the study period (2009–2023), the core case definition and reporting rules remained unchanged.

### 2.4 Statistical methods

#### 2.4.1 Joinpoint regression analysis

The Joinpoint regression model was selected to examine the OID incidence patterns in Qinghai Province due to its capability to detect significant trend inflection points. Identifying these inflection points is essential for comprehending the long-term, multi-phase dynamics of infectious diseases. The model applies a sequence of contiguous linear segments to the data, with each segment denoting a specific trend phase. The Grid Search Method (GSM) was utilized to ascertain the optimal quantity and positioning of these inflection points (22, 23). This method facilitates the computation of the Annual Percentage Change (APC), which quantifies the trend velocity inside each segment, and the Average Annual Percentage Change (AAPC), which encapsulates the overall trend velocity during the whole study period. A linear model was adopted for regularly distributed data, whereas a log-linear model was employed for data adhering to a Poisson or exponential distribution (24, 25). The ideal model was then determined by Monte Carlo permutation testing and the weighted Bayesian information criteria. The research utilized a comprehensive, population-based surveillance registry that exhibited a high level of data completeness throughout the study period; hence, no specific imputation for missing data was necessary. An APC over 0 signifies an upward trend during the period, while a negative APC indicates a downward trend. The model is articulated as follows:


$$\begin{array}{l} E_{\left(y|x\right)}=e^{\beta_{0}}+\beta_{1}x+\delta_{1}{\left(x-\tau_{1}\right)}^{+}+...{\;\delta \;}_{\text{k}}{\left(\text{x}-{\;\tau \;}_{\text{k}}\right)}^{+} \end{array}$$ (1)

$$\begin{array}{l} E_{\left(y|x\right)}=e^{\beta +\beta_{1}x+\delta_{1}{\left(x-\tau_{1}\right)}^{+}+...\;{\;\delta \;}_{\text{k}}{\left(\text{x}-{\;\tau \;}_{\text{k}}\right)}^{+}} \end{array}$$ (2)

Equation 1 pertains to the linear model, while Equation 2 pertains to the log-linear model; e denotes the base of the natural logarithm, β_0_ represents the intercept, and β_1_ signifies the regression coefficient. This study mainly used the log-linear model. The optimal number of joinpoints was determined using Monte Carlo permutation tests with a significance level of 0.05. A maximum of one joinpoint was allowed for all analyses due to the number of time points. The Grid Search Method (GSM) was utilized to ascertain the optimal quantity and positioning of inflection points. Joinpoint Regression Software (version 5.0.2; Surveillance Research Program, National Cancer Institute, Bethesda, MD, United States) was used with weighted least squares estimation assuming a Poisson distribution to account for heteroskedasticity arising from different population denominators.

#### 2.4.2 Spatial analysis

Moran's *I* index, encompassing both global and local versions, is a statistic that quantifies spatial autocorrelation among observations within a specified region. It is extensively utilized in medical statistics and various other domains for evaluating spatial dependence (26, 27). A Moran's *I* value of 1 signifies perfect positive spatial correlation, whereas a value of −1 implies perfect negative spatial correlation. This work analyzed global spatial autocorrelation using the global Moran's *I* index and visualized local spatial autocorrelation with LISA cluster maps (28). For both global and local Moran's *I* calculations, we constructed a first-order Queen contiguity spatial weights matrix (based on shared boundaries and vertices) with row standardization. Statistical significance was assessed using 499 Monte Carlo permutations. The spatial weights matrix was defined such that counties sharing a border or vertex were considered neighbors. Row standardization was applied to each weight matrix, meaning each neighbor weight was divided by the total number of neighbors for each county, ensuring that the sum of weights for each county equaled one. Raw incidence rates (per 100,000 population) were used directly without standardization, as Moran's *I* is scale-invariant and standardization does not affect the spatial autocorrelation coefficient. All spatial analyses were performed using ArcGIS Pro (version 3.0; Esri Inc., Redlands, CA, United States). Local cluster maps identified four types of spatial associations: high-high clusters (counties with high incidence surrounded by high-incidence neighbors), low-low clusters, high-low outliers, and low-high outliers. No separate hotspot analysis (e.g., Getis-Ord Gi^*^) was performed, as the study focused on identifying spatial autocorrelation patterns rather than detecting statistically significant hotspots of high values.

#### 2.4.3 Gravity center shift model and standard deviation ellipse

The standard deviational ellipse represents a classical methodology for characterizing the overall morphology of spatial disease distribution. It overcomes the limitation of the centroid shift model, which focuses exclusively on single-point movement. This approach utilizes four key parameters: the ellipse center, major axis, minor axis, and rotation angle (29). Together, these elements comprehensively reflect the concentration, spatial extent, and directional characteristics of the disease distribution. The incidence centroid for each year was calculated as shown in Equation (3)


$$\bar{X}=\frac{\Sigma_{n-1}^{n}w_{i}x_{i}}{\Sigma_{n-1}^{n}w_{i}},\bar{Y}=\frac{\Sigma_{i-1}^{n}w_{i}y_{i}}{\sum_{i-1}^{n}w_{i}}$$ (3)

where (*x*_*i*_, *y*_*i*_) are the coordinates of the centroid of county i, and *w*_*i*_ is the incidence rate of county i. The standard deviational ellipse parameters (center, major axis, minor axis, rotation angle) were calculated to describe the spatial distribution characteristics. The ellipse center aligns with the incidence centroid, the major axis indicates the primary direction of spatial spread, the minor axis reflects spatial dispersion in the perpendicular direction, and the rotation angle quantifies the orientation of the primary distribution axis. Interpretation of centroid migration reflects shifts in the spatial center of disease burden over time (30).

The ellipse center closely aligns with the incidence centroid of infectious diarrhea during the same period, representing a core spatial anchor for the disease. The major axis, defined as the longest diameter of the ellipse, indicates the primary direction of spatial spread (31, 32). A longer major axis signifies a wider diffusion range and greater dispersion along this direction. Conversely, the minor axis, as the shortest diameter, corresponds to the secondary direction of distribution, with its length inversely related to the compactness in that direction. A larger ratio of the major to minor axes suggests a more ribbon-like spatial pattern with pronounced directionality. The rotation angle, measured between the major axis and true north, directly quantifies the specific orientation of the primary distribution direction. These parameters are widely employed in analytical practice (33).

#### 2.4.4 Statistical analysis

Data regarding the incidence and mortality of OID in Qinghai Province were aggregated with Microsoft Excel 2019. A double-axis graph of incidence rates was produced utilizing Origin 2024. Joinpoint Regression Software (version 5.0.2) was employed to examine annual changes in OID incidence across different prefectures and age demographics in Qinghai Province, utilizing APC, AAPC, and their 95% confidence intervals (CIs) as primary metrics. Spatial analysis was performed with ArcGIS Pro (version 3.0). STL (Seasonal-Trend decomposition using Loess) was applied to monthly OID incidence data to disentangle the time series into trend, seasonal, and residual components, with the seasonal component assumed constant across years and the trend component extracted using locally weighted regression. This analysis was conducted using R software (version 4.5.1; R Foundation for Statistical Computing, Vienna, Austria).

## 3 Results

### 3.1 Epidemiological overview of OID in Qinghai Province

**Figure 1:**
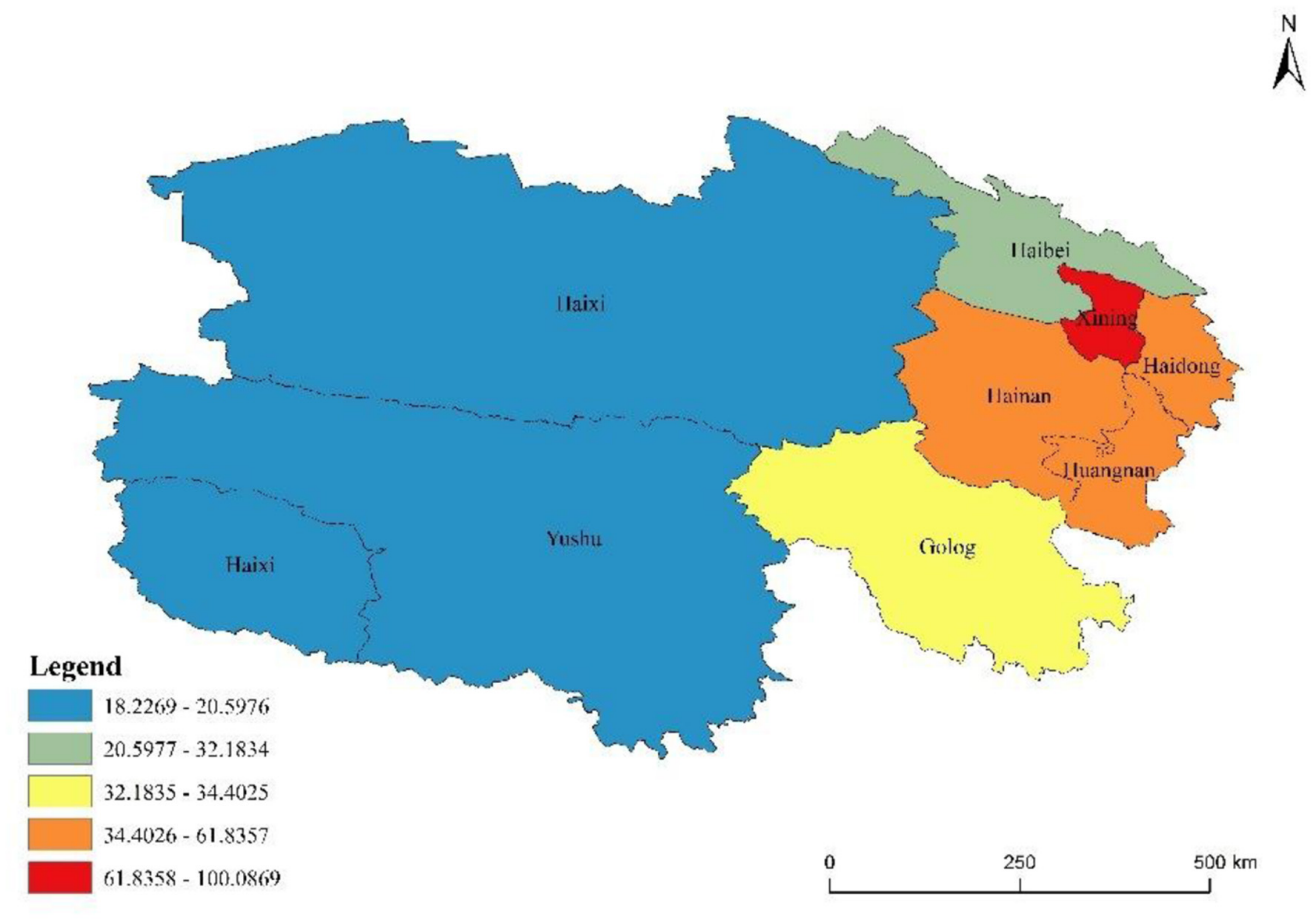
Annual average incidence rate of OID in Qinghai Province from 2009 to 2023.

**Figure 2:**
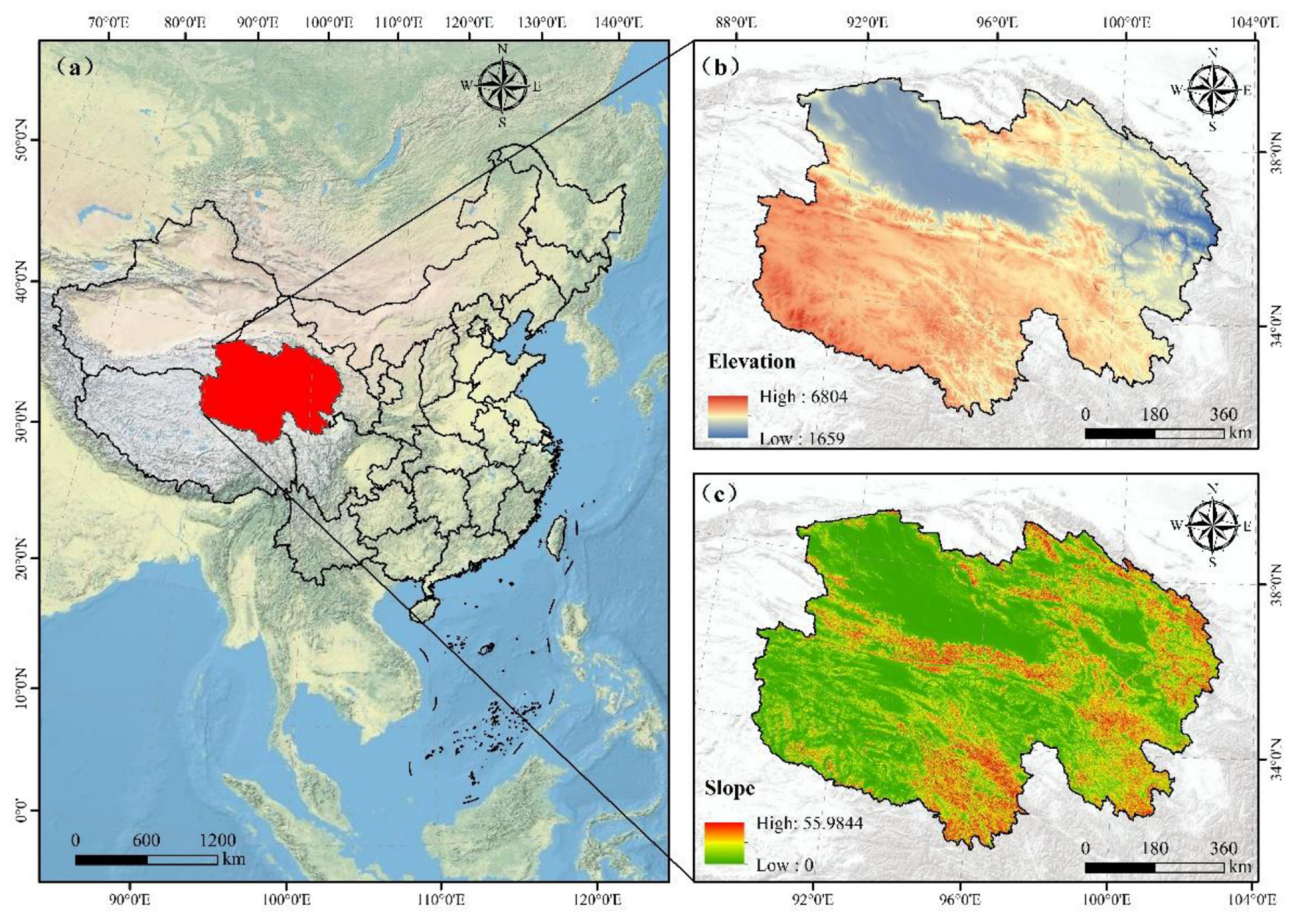
Geographical location and topographic characteristics of Qinghai Province, China. **(a)** Map of China showing the geographical location of Qinghai Province. **(b)** Elevation map of Qinghai Province with color-coded gradient from low to high elevation. **(c)** Slope map of Qinghai Province with color-coded gradient from low to high slope values.

From 2009 to 2023, a total of 58,717 cases of OID were reported in Qinghai Province, with an average annual incidence rate of 66.78 per 100,000 population calculated using mid-year permanent resident population estimates for each year. Only one fatal case was recorded, which occurred in 2020. According to surveillance data spanning 2009–2023, both the number of cases and the incidence rate of OID showed an overall upward trend, with a marked increase observed particularly after 2020. The number of reported cases increased from 1,688 in 2009 to 6,172 in 2023, and the incidence rate rose from 30.45 to 103.74 per 100,000 population, representing an increase of 240.69%. Detailed data are presented in Table 1 and Figure 1. And the specific location and altitude of Qinghai Province are shown in Figure 2.

**Table 1:** Incidence and mortality of OID in Qinghai Province from 2009 to 2023.

Year	Number of cases	Annual average incidence rate (/10^5^)	Number of deaths	Annual mortality rate (/10^5^)
2009	1,688	30.45	0	0.0000
2010	2,375	42.62	0	0.0000
2011	2,837	50.42	0	0.0000
2012	4,165	73.31	0	0.0000
2013	4,213	73.50	0	0.0000
2014	3,462	59.92	0	0.0000
2015	3,894	66.74	0	0.0000
2016	4,571	77.68	0	0.0000
2017	4,404	74.27	0	0.0000
2018	3,546	59.26	0	0.0000
2019	4,353	72.16	0	0.0000
2020	5,497	90.43	1	0.0165
2021	4,288	72.38	0	0.0000
2022	3,252	54.75	0	0.0000
2023	6,172	103.74	0	0.0000
2009–2023	58,717	66.78	1	0.0011

### 3.2 Temporal trends

#### 3.2.1 Annual trend analysis

As shown in Table 2, the incidence of OID in Qinghai Province showed a statistically significant overall upward trend from 2009 to 2023. The Joinpoint regression analysis identified the year 2012 as a statistically significant turning point (*p* < 0.05), which provided the objective basis for dividing the study period into two distinct temporal phases: 2009–2012 and 2012–2023. A period of rapid increase before 2012 was followed by a much slower rise in subsequent years.

**Table 2:** Joinpoint analysis of OID incidence in Qinghai Province from 2009 to 2023.

Variable	Year	APC value (95% CI)/%	AAPC value/%	*p*-Value
Male	2009–2012	26.84 (−15.11, 89.53)	5.94 (−2.26, 14.83)	0.160
	2012–2023	0.86 (−3.08, 4.97)		
Female	2009–2012	31.47 (−10.87, 93.93)	9.11 (1.03, 17.83)^*^	0.026
	2012–2023	3.69 (0.26, 7.26)^*^		
Overall	2009–2012	28.20 (4.07, 108.97)^*^	7.11 (2.03, 13.43)^*^	0.008
	2012–2023	1.98 (−16.95, 6.34)		

^*^The *p*-Value of APC is less than 0.05.

APC, Annual Percentage Change; AAPC, Average Annual Percentage Change.

The increasing trend was more pronounced and statistically significant in females, whereas the overall trend among males did not reach statistical significance. All genders exhibited a similar temporal pattern, characterized by a sharp rise before 2012 and a moderated increase thereafter, though the post-2012 trend remained significant only in females.

#### 3.2.2 Seasonal decomposition analysis

**Figure 3:**
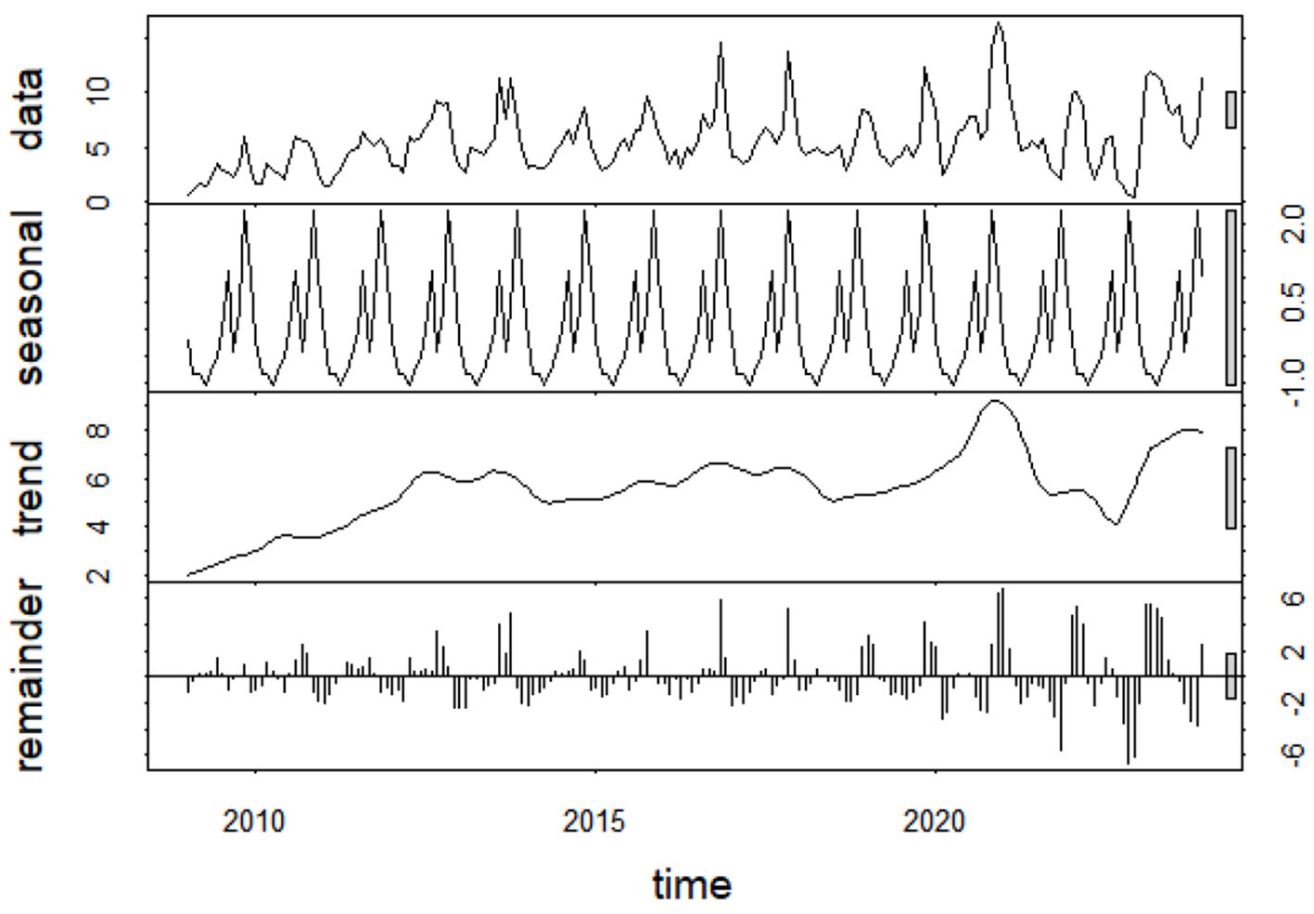
STL decomposition of monthly OID incidence in Qinghai Province, 2009–2023.

STL applied to monthly OID incidence data revealed distinct temporal components. The trend component showed an overall fluctuating increase from 2009 to 2019, peaking in 2019, followed by a slight decline, then rising again after 2020. The seasonal component demonstrated consistent annual periodicity, confirming the strong seasonal pattern of OID with winter peaks and summer nadirs. The residual component fluctuated randomly around zero, indicating that the trend and seasonal components captured the main information in the data (Figure 3).

### 3.3 Demographic patterns

#### 3.3.1 Joinpoint regression analysis of the overall incidence rate in Qinghai Province

Significant disparities in incidence levels and trends were observed across different regions. Xining City and Haidong City exhibited the highest average annual incidence rates, at 100.09 and 61.84 per 100,000 population, respectively. Among the autonomous prefectures, Huangnan Tibetan Autonomous Prefecture (52.44 per 100,000) and Hainan Tibetan Autonomous Prefecture (48.74 per 100,000) exhibited intermediate incidence levels. In contrast, the remaining prefectures reported comparatively lower rates, with Haibei Tibetan Autonomous Prefecture at 32.18 per 100,000, Golog Tibetan Autonomous Prefecture at 34.40 per 100,000, Haixi Mongolian and Tibetan Autonomous Prefecture at 20.60 per 100,000, and Yushu Tibetan Autonomous Prefecture at the lowest level of 18.23 per 100,000 (Table 3).

**Table 3:** Incidence and joinpoint analysis of various regions in Qinghai Province from 2009 to 2023.

Region	Annual average incidence rate (/10^5^)	Total number of cases	Section 1		Section 2		AAPC value/%	*p*-Value
			Year	APC value (95% CI)/%	Year	APC value (95% CI)/%		
Xining	100.09	34,925	2009–2012	31.13 (4.97, 114.47)^*^	2012–2023	0.61 (−14.81, 3.91)	6.49 (1.38, 12.65)	0.019
Haidong	61.84	13,311	2009–2012	21.83 (6.16, 53.77)^*^	2012–2023	3.22 (−6.09, 5.57)	6.95 (4.05, 9.85)	< 0.001
Haibei	32.18	1,349	2009–2012	43.45 (4.37, 282.02)^*^	2012–2023	9.13 (−24.67, 27.07)	15.71 (7.38, 31.46)	< 0.001
Huangnan	52.44	2,127	2009–2012	47.41 (0.89, 366.19)^*^	2012–2023	6.22 (−28.09, 26.78)	13.95 (4.26, 31.44)	< 0.001
Hainan	48.74	3,321	2009–2012	21.20 (−2.91, 78.28)	2012–2023	−0.28 (−19.09, 21.32)	3.98 (−0.93, 9.15)	0.129
Golog	34.40	1,036	2009–2021	8.80 (−34.55, 365.20)	2021–2023	35.83 (−11.76, 87.36)	12.30 (4.26, 37.76)	< 0.001
Yushu	18.23	1,101	2009–2021	1.71 (−33.65, 262.82)	2021–2023	43.55 (0.62, 94.47)^*^	6.84 (−1.38, 27.14)	0.103
Haixi	20.69	1,542	2009–2012	45.79 (−4.16, 382.19)	2012–2023	1.19 (−34.45, 20.29)	9.43 (−1.61, 27.17)	0.074

^*^The *p*-Value of APC is less than 0.05.

APC, Annual Percentage Change; AAPC, Average Annual Percentage Change.

Joinpoint regression analysis of OID incidence across various prefectures in Qinghai from 2009 to 2023 revealed an upward trend in the majority of regions, with AAPC values ranging from 3.98 to 15.71%. The most substantial increases were observed in Haibei Tibetan Autonomous Prefecture (AAPC = 15.71%, *p* < 0.001), Huangnan Tibetan Autonomous Prefecture (AAPC = 13.95%, *p* < 0.001), and Golog Tibetan Autonomous Prefecture (AAPC = 12.30%, *p* < 0.001).

Phased variation analysis indicated that most regions experienced a rapid increase in incidence between 2009 and 2012. Statistically significant APC values exceeding 20% were identified in Xining City, Haidong City, Haibei Tibetan Autonomous Prefecture, and Huangnan Tibetan Autonomous Prefecture (*p* < 0.05). From 2012 to 2023, the growth slowed or showed minor fluctuations in most regions, except for Golog and Yushu Tibetan Autonomous Prefectures. These two prefectures exhibited a resurgence in incidence during 2021–2023, with APC values reaching 35.83 and 43.55%, respectively.

#### 3.3.2 Incidence by age group and joinpoint regression analysis in Qinghai Province

From 2009 to 2023, marked disparities in incidence levels and long-term trends were observed across different age groups for OID in Qinghai Province. Annual incidence rates were characterized by an initial increase followed by a decrease with advancing age, with infants and the older adults representing high-risk populations. The highest average annual incidence rates were recorded in the 0~ < 5 year age group (890.45 per 100,000) and the 5~ < 10 year age group (46.92 per 100,000). After the age of 5 years, incidence declined sharply, remaining low throughout adolescence and adulthood. From the 40-year age group onward, a gradual resurgence was noted, with the incidence in the ≥80-year age group exceeding 25 per 100,000 again (Table 4).

**Table 4:** Incidence and joinpoint analysis of various age groups in Qinghai Province.

Region	Annual average incidence rate (/10^5^)	Total number of cases	Section 1		Section 2		AAPC value/%	*p*-Value
			Year	APC value (95% CI)/%	Year	APC value (95% CI)/%		
0~	890.45	49,811	2009–2012	36.66 (7.50, 130.08)	2012–2023	2.12 (−9.17, 5.68)	8.70 (3.69, 15.33)	0.008
5~	46.92	2,852	2009–2014	−3.48 (−35.65, 54.68)	2014–2023	5.88 (−27.41, 40.23)	2.43 (−2.74, 9.51)	0.338
10~	21.21	1,280	2009–2020	8.38 (−17.37, 200.06)	2020–2023	−18.37 (−62.82, 14.60)	1.99 (−7.07, 19.78)	0.418
15~	5.93	391	2009–2012	21.96 (−15.87, 233.45)	2012–2023	0.89 (−55.76, 44.72)	5.07 (−7.33, 19.92)	0.239
20~	5.31	360	2009–2011	36.21 (−7.33, 205.14)	2011–2023	3.03 (−39.18, 30.01)	7.47 (−2.08, 19.87)	0.098
25~	5.44	335	2009–2012	17.40 (−7.53, 115.30)	2012–2023	−0.71 (−37.20, 23.48)	2.91 (−4.61, 11.33)	0.334
30~	4.67	332	2009–2021	2.80 (−27.42, 88.71)	2021–2023	25.71 (−20.46, 69.75)	5.80 (−0.36, 14.59)	0.063
35~	3.94	308	2009–2021	1.20 (−24.85, 75.33)	2021–2023	29.77 (−12.09, 69.35)	4.86 (−1.37, 11.56)	0.118
40~	5.13	406	2009–2013	12.33 (1.69, 45.75)^*^	2013–2023	2.56 (−14.36, 12.78)	5.26 (2.05, 8.79)	< 0.001
45~	5.31	420	2009–2014	−2.65 (−30.12, 79.90)	2014–2023	2.17 (−33.87, 33.60)	0.42 (−5.00, 9.51)	0.800
50~	8.59	422	2009–2016	12.40 (2.31, 103.64)^*^	2016–2023	−7.86 (−31.55, −0.11)^*^	1.77 (−3.39, 10.94)	0.383
55~	7.96	347	2009–2013	17.58 (−16.43, 307.14)	2013–2023	−1.67 (−52.11, 39.79)	3.48 (−6.42, 23.26)	0.355
60~	10.38	308	2009–2013	12.01 (−2.97, 79.25)	2015–2023	−0.43 (−29.77, 12.06)	2.98 (−2.28, 9.07)	0.167
65~	13.21	340	2009–2015	5.59 (−11.60, 72.14)	2015–2023	−3.18 (−33.67, 16.23)	0.49 (−4.78, 7.27)	0.792
70~	16.92	329	2009–2021	−0.10 (−28.38, 67.96)	2021–2023	18.90 (−14.02, 55.18)	2.41 (−3.12, 8.98)	0.424
75~	22.03	275	2009–2019	6.10 (−9.21, 113.81)	2019–2023	−7.86 (−47.94, 10.19)	1.92 (−4.22, 13.12)	0.328
80~	25.32	140	2009–2019	5.22 (0.17, 65.09)^*^	2019–2023	−16.32 (−44.24, −1.63)^*^	−1.45 (−6.50, 7.52)	0.756
≥85	26.49	61	2009–2011	−39.60 (−70.84, 28.95)	2011–2023	−0.45 (−52.51, 70.52)	−7.31 (−13.80, 6.31)	0.194

^*^The p-Value of APC is less than 0.05.

APC, Annual Percentage Change; AAPC, Average Annual Percentage Change.

Joinpoint regression analysis indicated an upward trend in incidence in most age groups during 2009–2023. The highest AAPC values were observed among children aged 1–15 years, with the most pronounced increases in the 0-year (AAPC = 8.70%, *p* = 0.008), 20-year (AAPC = 7.47%, *p* = 0.098), and 30-year (AAPC = 5.80%, *p* = 0.063) age groups. In contrast, negative AAPC values were found in the 80- and ≥85-year age groups, though these trends were not statistically significant.

### 3.4 Spatial autocorrelation analysis

#### 3.4.1 Global spatial autocorrelation analysis

As presented in Table 5, a significant positive global spatial autocorrelation was observed in the incidence of OID in Qinghai Province across all 15 consecutive years of the study (all *p*-values < 0.05). Specifically, Moran's *I* values for each year were positive, ranging from 0.4042 to 0.6432. The highest Moran's *I* value was recorded in 2016 (*I* = 0.6432, *Z* = 7.2540, *p* < 0.0001), indicating the strongest spatial clustering in that year. Although some fluctuations were noted between years—such as decreases in 2014 and 2018—the overall Moran's *I* values remained high. In addition, all *Z*-scores were large and statistically significant (all *p*-values < 0.001). These results demonstrate that the spatial distribution of OID in Qinghai Province was not random but rather exhibited significant spatial dependence and heterogeneity. However, no covariates were included in the spatial analysis; therefore, the interpretation of spatial patterns is descriptive and does not account for potential confounding factors such as socioeconomic or environmental variables.

**Table 5:** Global autocorrelation analysis of OID in Qinghai Province.

Year	Moran's *I* value	*Z* value	*p-*Value
2009	0.4518	5.1209	< 0.0001
2010	0.5253	6.0169	< 0.0001
2011	0.5950	6.5960	< 0.0001
2012	0.5383	6.0503	< 0.0001
2013	0.6123	6.8611	< 0.0001
2014	0.5739	6.5020	< 0.0001
2015	0.5779	6.4693	< 0.0001
2016	0.6432	7.2540	< 0.0001
2017	0.6235	7.1062	< 0.0001
2018	0.4704	5.2837	< 0.0001
2019	0.5344	6.0029	< 0.0001
2020	0.5555	6.3149	< 0.0001
2021	0.5553	6.1016	< 0.0001
2022	0.4507	5.0199	< 0.0001
2023	0.4042	4.5192	< 0.0001

#### 3.4.2 Local spatial autocorrelation analysis

**Figure 4:**
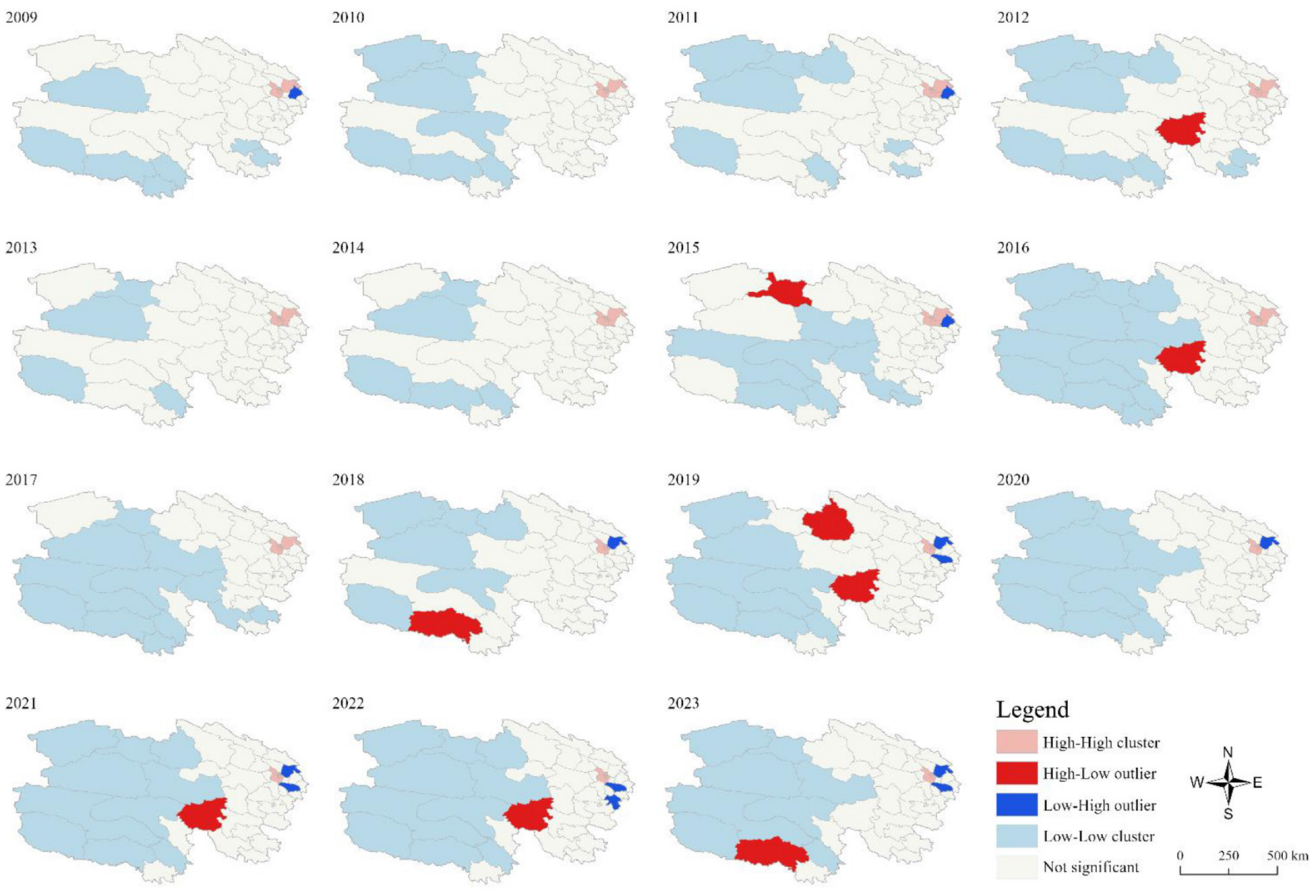
LASA aggregation map of OID in Qinghai Province from 2009 to 2023.

Local spatial autocorrelation analysis identified clusters of high-high aggregation in Xining City and Haidong City from 2009 to 2023. In contrast, low-low clusters were observed in Yushu Tibetan Autonomous Prefecture and Haixi Mongolian and Tibetan Autonomous Prefecture. These results indicate clear regional variation in the spatial distribution of OID across Qinghai Province (see Figure 4). And to assess robustness, sensitivity analyses were conducted at the prefecture level rather than the county level, which yielded consistent spatial clustering patterns with Xining and Haidong remaining as high-high clusters.

#### 3.4.3 Standard deviation ellipse analysis and gravity center migration

From 2009 to 2023, the incidence of other infectious diarrhea in Qinghai Province was characterized by a primary clustering region centered around Xining and Haidong cities. The overall spatial pattern was oriented in a northeast-southwest direction, indicating this as the dominant axis of spatial spread. This pattern was elucidated through standard deviational ellipse analysis. Across all study years, the long axis of the ellipse was consistently and markedly longer than the short axis. This finding demonstrates a broader distribution and higher spatial dispersion along the northeast-southwest axis, while the distribution was relatively more concentrated in the northwest-southeast direction. This disparity in axial length reflects a distinct spatial heterogeneity driven by the Xining core. Although the ellipse morphology exhibited periodic fluctuations over the years, indicating alternating expansion and contraction of the spatial range, the core cluster remained consistently stable around Xining without significant regional shift.

An analysis of the centroid migration trajectory revealed that the incidence centroid was consistently located in the area south of Xining city. The migration path was delineated by a distinct three-phase pattern: stable fluctuation-significant shift-gradual return. From 2009 to 2019, the centroid fluctuated within a small area around Xining, suggesting a relatively stable spatial pattern. Between 2019 and 2021, a marked southwestward jump in the centroid was observed, representing the largest migratory amplitude in the study period. This shift reflected a temporary expansion of the core cluster toward the southwest. From 2021 to 2023, the centroid gradually shifted northwestward, returning to the vicinity of the early-stage distribution area around Xining. This trajectory indicated a dynamic adjustment and a return trend toward the established core region.

**Figure 5:**
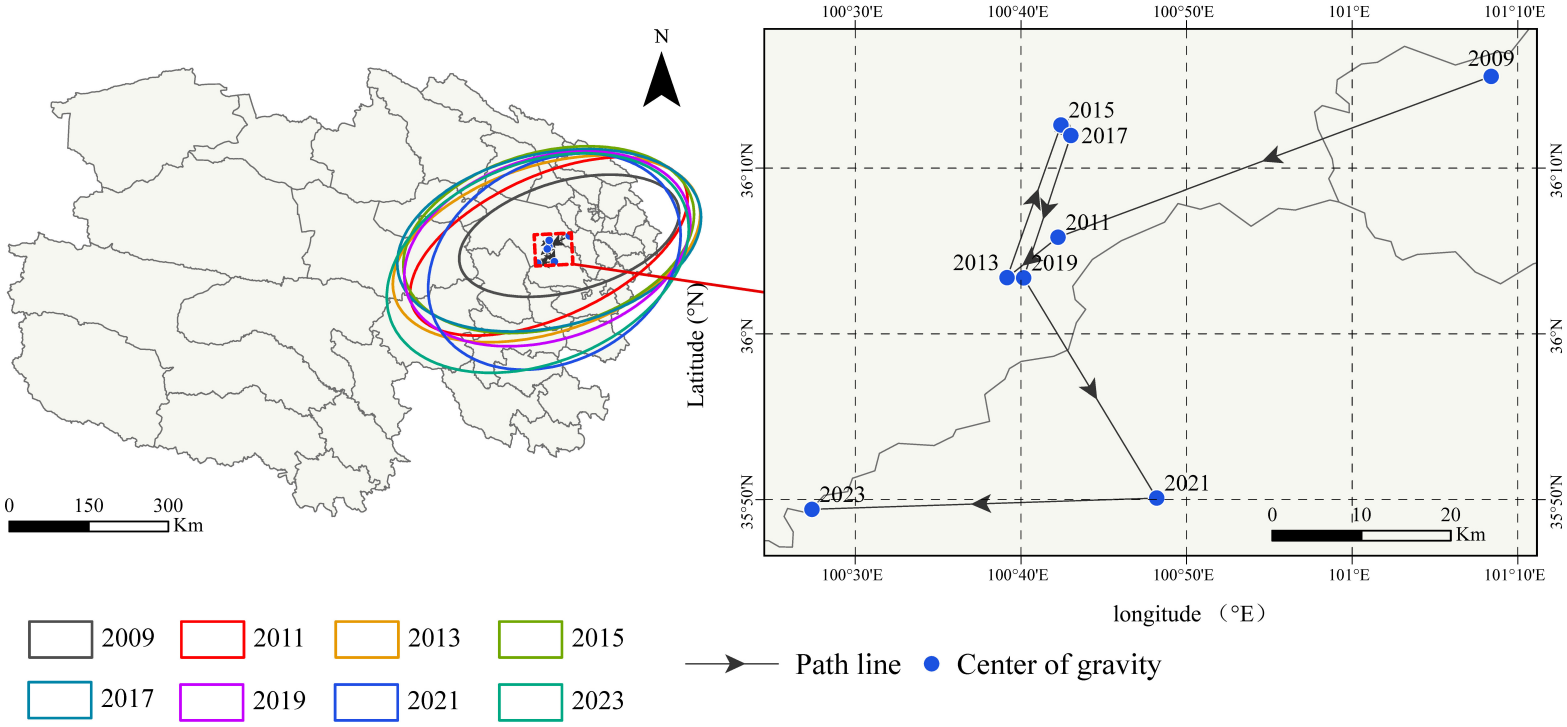
The gravity center of OID incidence from 2009 to 2023 in Qinghai.

By integrating the ellipse morphology with the centroid evolution, the spatial distribution of incidence from 2009 to 2023 can be characterized by two phases. The period of 2009–2019 was a stable phase, during which the ellipse morphology showed minimal fluctuation and the centroid remained stable around Xining. The period of 2019–2023 was a transition phase, characterized by a significant southwestward centroid shift and corresponding adjustments in ellipse morphology, followed by a gradual northwestward return to the Xining-centered core area. For further details (see Figure 5).

## 4 Discussion

This study provides the first comprehensive spatiotemporal analysis of other infectious diarrhea in Qinghai's high-altitude setting, revealing a significant increase in incidence from 2009 to 2023 (AAPC = 7.11%, *p* = 0.008), with marked spatial clustering in the eastern urban corridor (Xining and Haidong) and a bimodal age distribution peaking in children under five and the older adults.

The temporal trend analysis identified 2012 as a critical turning point in OID incidence in Qinghai Province. The rapid increase during 2009–2012 (APC = 28.20%) likely reflects enhanced surveillance sensitivity through the establishment of foodborne disease sentinel networks in 2012, combined with active circulation of pathogens such as rotavirus. Subsequently, the slower growth phase from 2012 to 2023 (APC = 1.98%) suggests the effectiveness of public health interventions, particularly the widespread rotavirus vaccination that substantially reduced incidence among young children.

From 2009 to 2023, the increase in incidence was greater among females (AAPC = 9.11%, *p* = 0.026) than males (AAPC = 5.94%, *p* = 0.160). Whether this disparity is linked to differential household exposure or healthcare-seeking behaviors warrants further investigation, a pattern that aligns with findings from other Chinese provinces such as Hunan (34–36). Biologically, sex-based differences in immune response to enteric pathogens have been documented, with females typically exhibiting stronger immune responses that may paradoxically increase susceptibility to inflammatory sequelae. Socially, women in Qinghai's predominantly agricultural and pastoral communities often assume greater responsibility for childcare, food preparation, and household water management, potentially increasing their exposure to enteric pathogens. Additionally, healthcare-seeking behavior may differ by sex, with women more likely to seek care for acute illnesses, potentially leading to higher reporting rates. These hypotheses require further investigation through behavioral and exposure assessment studies.

Spatially, the incidence of OID in Qinghai Province exhibited significant heterogeneity. Xining City and Haidong City, which are relatively developed and densely populated, reported the highest average annual incidence rates (100.09 and 100.68 per 100,000, respectively), suggesting a potential association among urbanization, population mobility, and disease risk (37–39). However, prefectures such as Haibei, Huangnan, and Golog, which had relatively lower average incidence rates, showed significant and rapid increasing trends (all AAPC > 12%, *p* < 0.001). These findings indicate distinct public health priorities: in high-incidence urban centers, targeted interventions should focus on improving environmental sanitation, food safety, and daycare center management, whereas in rapidly emerging regions like Haibei, Huangnan, and Golog, efforts should prioritize enhancing primary care diagnostic capacity and community-based surveillance to contain outbreaks before they escalate (40, 41).

Age-specific distribution analysis revealed a consistent bimodal pattern, with the highest incidence concentrated in infants and young children (0– < 5 years) and the older adults (≥80 years). Notably, the 1-4-year-old group not only had a high baseline risk but also experienced the most rapid increase over the study period (AAPC > 10%, *p* < 0.001). This underscores the need for life-course preventive strategies. For young children, this includes promoting exclusive breastfeeding, strengthening rotavirus vaccination coverage, and providing targeted hygiene education in kindergartens. For the older adults, especially those with chronic conditions, interventions should focus on improving in-home care, ensuring safe drinking water, and raising awareness of early symptom recognition (42–44).

Spatial autocorrelation analysis confirmed that OID cases were not distributed randomly but exhibited stable spatial clustering. Global Moran's *I* values were consistently positive (0.404–0.643, all *p* < 0.05), indicating significant positive spatial autocorrelation at the global scale. Local autocorrelation identified high-high clusters mainly within Xining and Haidong cities. This spatial pattern may be related to regional disparities in healthcare accessibility, population density, and health education coverage (45–47). Targeted inspection and improvement of rural water supplies and urban water distribution networks in clustered areas are recommended to ensure comprehensive drinking water treatment and disinfection (48, 49).

The analysis revealed a strongly aggregated and spatially stable distribution of OID in Qinghai Province from 2009 to 2023, consistently concentrated in the eastern core region with no shift to remote western or southern areas. This pattern was closely associated with high population density, urbanization, population mobility, and sanitation infrastructure limitations, while enhanced disease reporting sensitivity further reinforced the observed aggregation (50). The evolutionary process comprised three phases: initial expansion and eastward movement linked to surveillance network improvements, subsequent contraction and stabilization likely due to targeted interventions, and recent southeastward shift associated with post-pandemic mobility recovery and population influx. Consequently, prevention strategies should focus on sustained management in the eastern core region, including enhanced drinking water monitoring, institutional prevention measures, and dynamic cross-county coordination mechanisms (51).

This study presents a systematic analysis of OID characteristics in Qinghai's high-altitude setting, providing valuable insights into diarrheal disease transmission under special geographical conditions. Our findings identify clear spatial clusters centered in Xining and Haidong cities, along with vulnerable populations such as young children and older adults individuals. These results not only inform region-specific public health planning within Qinghai but also contribute to the broader understanding of diarrheal disease epidemiology in high-altitude regions globally, where similar environmental constraints may influence transmission dynamics. The observed regional variations in incidence patterns highlight the need for tailored prevention strategies and efficient resource allocation across different areas. Moreover, the integrated analytical framework developed in this research, which combines temporal decomposition, spatial autocorrelation, standard deviational ellipse, and gravity center shift modeling, offers a methodological reference for similar studies in other high-altitude regions, such as the Andean Plateau or the Himalayan belt. The empirical evidence from this study thus contributes to formulating science-based control guidelines while generating hypotheses for future comparative and mechanistic investigations across diverse geographic settings.

## 5 Conclusion

In summary, OID incidence in Qinghai Province increased significantly from 2009 to 2023, with distinct spatiotemporal and demographic patterns. The persistent high-high clusters in Xining and Haidong warrant enhanced environmental sanitation and food safety inspections in urban areas. The rapid increases in Haibei, Huangnan, and Golog call for strengthening primary healthcare diagnostic capacity and community-based surveillance. The bimodal age distribution supports prioritizing rotavirus vaccination for children and targeted hygiene education for households with older adults members. Standard deviational ellipse analysis revealed a stable core cluster centered on Xining and Haidong, underscoring the need for sustained cross-county coordination in diarrheal disease control efforts within this region.

## 6 Limitations to address

Several limitations of this study should be noted. First, passive surveillance data may involve underreporting or misclassification, with reporting completeness varying across counties due to differences in healthcare access and diagnostic capacity. Second, the ecological study design precludes individual-level inferences, and observed county-level associations may not reflect individual risk factors (ecological fallacy). Third, environmental and socioeconomic covariates were not included, limiting exploration of driving factors. Fourth, county-level analysis may mask within-county heterogeneity. Fifth, crude incidence rates were used without age-standardization, limiting comparability across regions with different age structures. Finally, the spatial autocorrelation analysis assumed a fixed spatial weight matrix, which may not capture temporal changes in spatial relationships.

## Data Availability

The raw data supporting the conclusions of this article will be made available by the authors, without undue reservation.
